# Effects of Cannabis Use on the Protein and Lipid Profile of Olfactory Neuroepithelium Cells from Schizophrenia Patients Studied by Synchrotron-Based FTIR Spectroscopy

**DOI:** 10.3390/biom10020329

**Published:** 2020-02-19

**Authors:** Sergi Saladrigas-Manjón, Tanja Dučić, Liliana Galindo, Cristina Fernández-Avilés, Víctor Pérez, Rafael de la Torre, Patricia Robledo

**Affiliations:** 1 Integrative Pharmacology and Systems Neuroscience, IMIM-Hospital del Mar Research Institute, 08003 Barcelona, Spain; sergi.saladrigas75@gmail.com (S.S.-M.); cfernandez@imim.es (C.F.-A.); rtorre2@imim.es (R.d.l.T.); 2 ALBA-CELLS Synchrotron, MIRAS Beamline, 08290 Cerdanyola del Vallès, Spain; tducic@cells.es; 3 Neuropsychiatry and Addictions Institute (INAD) of Parc de Salut Mar, 08003 Barcelona, Spain; liligalindo@gmail.com (L.G.); 61155@parcdesalutmar.cat (V.P.); 4 Department of Psychiatry, University of Cambridge, Cambridge CB2 0SZ, UK; 5 CIBERSAM and Department of Psychiatry, University Autonoma of Barcelona, 08193 Barcelona, Spain

**Keywords:** schizophrenia, olfactory neuroepithelium, SR-FTIR, cannabis

## Abstract

Schizophrenia (SCZ) is a neurodevelopmental disorder with a high genetic component, but the presence of environmental stressors can be important for its onset and progression. Cannabis use can be a major risk factor for developing SCZ. However, despite the available data on the neurobiological underpinnings of SCZ, there is an important lack of studies in human neuronal tissue and living cells addressing the effects of cannabis in SCZ patients. In this study, we analysed the most relevant bio-macromolecular constituents in olfactory neuroepithelium (ON) cells of healthy controls non-cannabis users, healthy cannabis users, SCZ patients non-cannabis users, and SCZ patients cannabis users using Synchrotron Radiation-Fourier Transform Infrared (SR-FTIR) spectrometry and microscopy. Our results revealed that SCZ patients non-cannabis users, and healthy cannabis users exhibit similar alterations in the macromolecular profile of ON cells, including disruption in lipid composition, increased lipid membrane renewal rate and lipid peroxidation, altered proteins containing more β-sheet structures, and showed an increase in DNA and histone methylation. Notably, these alterations were not observed in SCZ patients who use cannabis regularly. These data suggest a differential effect of cannabis in healthy controls and in SCZ patients in terms of the macromolecular constituents of ON cells.

## 1. Introduction

Schizophrenia (SCZ) is a highly prevalent chronic and severe neuropsychiatric disorder. According to the World Health Organization [1], it is estimated to affect more than 21 million people worldwide. Socially, it clearly causes a familiar and economic burden, and patients’ lives are shadowed by social stereotypes and stigmas [2]. SCZ is thought to be a neurodevelopmental disorder with a high genetic component [3]. Genetic studies have suggested several potential affected genes, such as Disc1 (disrupted-in-schizophrenia 1), Nrg1 (Neuregulin-1), or DAO (Diamine Oxidase), and other regions of linkage and positional associated chromosomal abnormalities [4]. The gene Disc1 has been shown to contribute to SCZ onset [5], and in animal models, it has been reported that Disc1 protein aggregations, predominantly in GABAergic prefrontal interneurons are related to SCZ-like symptoms [6]. In addition, many changes in lipid membrane composition and renewal rates [7,8], and lipid metabolism, such as peroxidation [9], have been described in SCZ patients’ blood serum. Although there are still important issues regarding the use of common proteomic platforms, and sample processing, the data point towards major changes in cytoskeleton and metabolic pathways. In addition, some of the most prominent changes in SCZ have been found in glial proteins [10].

Environmental factors can play a crucial role in the development of SCZ. Thus, the double hit hypothesis states that genetic factors together with the presence of one or several environmental cues are necessary for the disease to initiate its chronic course [11]. In this sense, the highly prevalent consumption of drugs of abuse in the SCZ population, particularly cannabis, could impact the onset, development, and chronicity of the disease [12]. *Cannabis sativa* is a recreational drug widely consumed in Europe, especially during the ages where SCZ onset usually occurs namely, late adolescence and early adulthood [13]. Chronic cannabis use alters cognitive, rewarding and affective processes [14,15,16] that are also critically altered in SCZ, by acting on the endocannabinoid system, and by modulating the activity of other neurotransmitters in the brain. The endocannabinoid system (ECS) consists of two major G protein-coupled receptors: the cannabinoid type 1 (CB_1_R) and type 2 (CB_2_R) receptors, two main endogenous ligands: anandamide (AEA) and 2-arachidonoylglycerol (2-AG) called endocannabinoids, and their synthetizing and degrading enzymes [17]. Despite the many preclinical, genetic and neuroimaging data that have been generated, the neuropathophysiological processes that lead to SCZ onset still remain unknown [18], and there are very few reports investigating the effects of cannabis use in sub-populations of SCZ patients that use cannabis regularly.

Obtaining viable neurological tissue to study how biomarkers of disease are modulated by drugs such as cannabis in living patients has proven challenging. In this sense, the olfactory neuroepithelium (ON) has great potential as a surrogate model of central nervous system function to study the molecular processes involved in some neuropsychiatric disorders such as SCZ [19]. Human olfactory sensory neurons are replaced and generated by neurogenesis continuously, throughout all the adult life, from neuronal precursors located in the apical and basal membranes [20]. Consequently, the ON contains pluripotent cells that can be easily and non-invasively collected and can proliferate in vitro and differentiate into multiple cell types, including neurons and glia [21]. Therefore, the use of this pro-neuronal tissue may open a very promising window to study neuropsychiatric disorders, and to determine the impact of drugs of abuse, including cannabis. In fact, we have recently demonstrated that ON cells obtained from cannabis users exhibit changes in cannabinoid CB_1_R and serotonin 2A receptor (CB_1_R-5HT_2A_R) heteromer expression and function, which were associated with cognitive performance [22].

In this study, we investigated the macromolecular profile of proteins and lipids of ON cells using infrared spectroscopy. Imaging of human single cells based on this technique is being progressively developed for biomedical applications. The spectral signature of a cell is a mirror image of its physiological status, and its reaction to environmental cues [23]. In order to achieve single cells resolution and spectral stability, we used Synchrotron Radiation-Fourier transformed infrared (SR-FTIR) spectroscopy at MIRAS beamline at ALBA Synchrotron. This non-destructive IR spectroscopy and imaging has a ~1000-fold higher brightness than conventional global sources, thus, by improving the signal-to-noise ratio, it is very useful for the analysis of single cells [24]. Moreover, SR-FTIR is able to provide information regarding the quantity, composition, and distribution of chemical functional groups including nucleic acids, lipids and proteins in stain-free and label-free samples [25].

Therefore, we compared the IR spectral profile of ON cells derived from SCZ patients non-cannabis users (SCZ/nc), and SCZ patients cannabis users (SCZ/c) with those of healthy controls non-cannabis users (HC/nc), and healthy controls cannabis users (HC/c).

## 2. Material and Methods

### 2.1. Subjects and Study Design

A cross-sectional study was conducted on 10 SCZ patients and 9 healthy subjects. These groups were further divided into subjects who use cannabis and subjects who do not use cannabis on a regular basis: HC/nc, HC/c, SCZ/nc and SCZ/c. The study was reviewed and approved by the local institutional ethics committee: “Clinical Research Ethical Committee of the Parc de Salut Mar, Barcelona, Spain (CEIC-PSMAR), on the 17^th^ of November 2014 (Ref. 2014/5801/l). All subjects provided a signed informed consent after a complete description of the study and procedures and were assured of the confidentiality of the data being collected. SCZ patients were recruited from the Institute of Neuropsychiatry and Addictions (INAD) in Barcelona. Eligible patients were 18 to 50 years of age with a primary psychiatric diagnosis of SCZ confirmed by the Structured Clinical Interview for DSM-IV-TR Axis I Disorders, Clinical Trial Version (SCID-CT) [26]. The exclusion criteria for SCZ patients were: (i) exhibit any comorbid axis I or II psychiatric condition; (ii) a current or previous diagnosis of substance use disorder, (iii) have received electroconvulsive therapy; (iv) present a medical condition that could affect nasal epithelia, including allergic rhinitis and other otorhinolaryngology conditions; (v) have history of severe neurological, congenital or medical condition; (vi) have allergy to the anaesthetic drug lidocaine and (vii) present severe negative or cognitive symptoms, educational level or language barrier that might prevent the subjects from possessing the necessary capacities to communicate and/or perform the evaluations tests. Healthy subjects matched by age and gender were recruited via oral or written advertising and underwent the same structured diagnostic interview to confirm eligibility. Healthy subjects fulfilled the following inclusion criteria: subjects of both sexes and age between 18 and 50 years were free of any axis I disorder in their familiar history (first- and second-degree relatives), and in the case of cannabis users, consumption of more than 5 cannabis cigarettes per week for more than 6 months. Cannabis users from both groups were instructed to interrupt cannabis use for at least 12 h before testing so as to avoid a potential confound of acute cannabis intoxication on the biochemical assessments.

### 2.2. Clinical and Functional Assessment

Subjects first underwent an initial evaluation that consisted of a complete physical examination, including body mass index calculation (BMI = weight in kg/height in m^2^), collection of medical and psychiatric personal and family history along with sociodemographic data. All the assessments and rating scales were conducted by experienced psychiatrists and psychiatry residents, as previously described by Galindo et al. [22]. Socioeconomic status (SES) was measured by the Hollingshead–Redlich Scale [27], whose score is derived from both education attainment and an occupation prestige. SES was then calculated as ([Occupation score × 5] + [Education score × 3]), with higher scores reflecting lower SES. Functionality level and social disability were cross-sectionally measured with the Global Assessment of Functioning (GAF) disability scale from the DSM-IV manual. Substance-related disorders were assessed with the Spanish validated version of the Psychiatric Research Interview for Substance and Mental Disorders (PRISM) [28].

### 2.3. Nasal Exfoliation and Cell Culture

Samples of the olfactory neuroepithelium (ON) were obtained from subjects during the morning, as previously described [22,29]. Before the nasal exfoliation, all participants were evaluated and instructed not to fast that morning. After humidification of the nasal cavity and anaesthesia with Lidocaine (Xilonibsa Aerosol^®^ 10 mg), separate sterile interdental brushes (two for each nostril) were used to obtain samples from the upper and middle turbinate. Samples were placed inside Eppendorf tubes with 250 μL of Dulbecco’s Modified Eagle Medium/ Ham F-12 (DMEM/F12) containing 10% FBS, 2% glutamine and 1% streptomycin-penicillin (GibcoBRL) at 4 °C. Cell suspensions were dissociated by mechanical disaggregation. The primary cultures were grown for 3 weeks in DMEM/F12 supplemented with 10% FBS at 37 °C and 5% CO_2_ before passaging into flasks (Thermo Scientific, Madrid, Spain). Primary cultures were dissociated with 0.25% trypsin (GibcoBRL), replated at 4000 cells/cm^2^ into 75 cm^2^ flasks and cultured in DMEM/F12 with 10% FBS. Cells were then expanded by passage and banked down in aliquots after harvest, followed by storage in liquid nitrogen with 20% FBS and 10% dimethyl sulfoxide (Sigma-Aldrich, Madrid, Spain). The ON cells used for the SR-FTIR spectroscopy analyses were frozen aliquots at passage 3 at a concentration of 1 × 10^6^ cells/mL. We added 20 μL from these aliquots in the centre of a CaF_2_ polished window (CRYSTAN Ltd.) submerged into a well with 500 μL of medium, thus 20,000 cells were finally plated in each window. One single window was plated for the cells of each subject. Samples were placed in the incubator during 24 h at 37 °C and 5% of CO_2_ in order to endure proper adherence of the cells. Afterwards, the medium was removed, cells were washed with saline and the windows were placed inside the refrigerator, letting them dry overnight. Finally, we placed the samples over silica gel to avoid humidity that could potentially produce interference in our measurements. CaF_2_ polished windows have some optical special properties that make them the most suitable surface to analyse dried cells with synchrotron infrared light transmission spectroscopy without interference [30].

### 2.4. Synchrotron-Radiation Fourier Transform Infrared (SR-FTIR) Spectroscopy

The SR-FTIR measurements were performed at the FTIR spectro-microscopy facility of MIRAS beamline at the synchrotron ALBA, in Cerdanyola del Vallès, Barcelona, Spain. The IR spectroscopic absorption measurements were collected in transmission mode using an IR microscope coupled to a SR-FTIR Hyperion 3000 spectrometer and microscope (Bruker, Ettlingen, Germany). The IR microscope was equipped with an automated sample stage, which could be controlled so as to define which cells had to be measured. The aperture was set to a 10 µm × 10 µm lighted by the infrared light and each spectrum was acquired after 256 scans at spectral resolution 4 cm^−1^. A mercury cadmium telluride (MCT) detector cooled with liquid nitrogen was used for collecting spectra. The infrared signal from synchrotron light used during the measurements was always at an optimal level of absorption so that detection of the macromolecular changes of the sample was ensured. The IR spectroscopic measurements were collected using the 36x objective in the IR transmission. In every sample, 20 to 32 cells were measured, and two measurements in each cell. All the regions measured were selected after using the online visible light microscope. All the settings were applied using OPUS 7.5 (developed by Bruker, Ettlingen, Germany), the software package with which Hyperion 3000 works.

### 2.5. Statistical Analyses

For the demographic categorical data (gender and tobacco use), differences between groups were assessed using two-tailed chi-squared test. Subsequently, to determine whether the different demographic, clinical, and functional data categories showed a normal distribution, Kolmogorov-Smirnov tests were first applied. Non-parametric Kruskal–Wallis test was used to assess differences in continuous variables that did not meet the assumption of a normal distribution. When significant interactions were found, individual non-parametric comparisons for each pair were performed using the non-parametric Dunn’s post-hoc test. The non-parametric Mann Whitney test was performed for differences between two groups and when normality was not assumed. Differences in variables with a normal distribution were assessed with one-way ANOVA tests and, when significant, comparison between groups was carried out by using LSD post-hoc test. Demographic, clinical and functional data were analysed with the PASW Statistics v.18 (3. SPSS Inc (2009): PASW Statistics for Windows (Version 18.0). P-values lower than 0.05 were considered statistically significant.

The principal component analysis (PCA) was performed by Unscrambler X, Version 10.3, (CAMO Software, Oslo, Norway) software packages. Next, using Unscrambler X, baseline correction and unit vector normalization was applied to the spectra. Without further corrections, a PCA was performed and calculated using the Nonlinear Iterative Partial Least Squares (NIPALS) algorithm on mean centred data, without applying any weighting method. PCA analysis showed descriptive information regarding where to find the major differences in the SR-FTIR spectra of the samples. Differences between groups in the major peaks of the lipid (3016 cm^−1^, 2956 cm^−1^, 2923 cm^−1^, 2871 cm^−1^, 2852 cm^−1^) and the fingerprint region (1652 cm^−1^, 1538 cm^−1^, 1081 cm^−1^, 1240 cm^−1^) were assessed using one-way ANOVA tests, and when necessary, statistical analysis was followed by the LSD post-hoc test. In addition, since, in the PCA analysis, differences in many hidden peaks were also found, two further analyses were performed. In order to avoid any human mistake in the interpretation of these results, we decided to exclude all the outliers of the lipid and protein regions, found in the PCA, from the statistical analysis.

First, the Norris Gap Derivative was applied in some intervals of the spectra using Unscrambler X (CAMO Software, Oslo, Norway), where prominent differences were found in the PCA analysis (such as around 2936 cm^−1^ spectra region). By using this technique, the second derivative of the spectra is obtained, showing hidden peaks, which can also be tested and could, explain reliable and important information that was missing before this last smoothing. These hidden peaks were analysed between groups by using one-way ANOVA tests, followed by LSD post-hoc tests when interaction was found. All the spectroscopy data was analysed with the PASW Statistics v.18 (3. SPSS Inc (2009): PASW Statistics for Windows (Version 18.0). P-values lower than 0.05 were considered statistically significant.

Second, a deconvolution of the Amide I area (1700 cm^−1^ to 1600 cm^−1^) of SR-FTIR spectra was carried out by using least-squares iterative curve fitting to Gaussian line shapes in OriginPro, Version 2019 software (OriginLab Corporation, Northampton, MA, USA). The deconvolution showed up specific secondary protein structure peaks that contributes to the Amide I major peak. Since secondary protein structures are not centred in just one specific wavenumber, but their contributions exist in a larger area of the spectra, the integrated area (expressed in percentage out of the total) of each secondary structure was calculated after the deconvolution, and then compared between groups using one-way ANOVA test, followed when necessary by LSD post-hoc test. These tests were carried out with the PASW Statistics v.18 (3. SPSS Inc (2009): PASW Statistics for Windows, Version 18.0, New York, NY, USA). P-values lower than 0.05 were considered statistically significant.

## 3. Results

### 3.1. Demographic, Socioeconomic, and Functional Characteristics of the Sample

The demographic, socioeconomic and functional data from all subjects are shown in Table 1. Briefly, groups did not differ in age, gender, history of nasal trauma, surgery, rhinitis or BMI. Socioeconomic status was not significantly different between groups. In contrast, differences in GAF scores were observed between groups (K = 14.60; *p* < 0.01), with SCZ/nc (*p* < 0.05) and SCZ/c (*p* < 0.05) showing significantly lower GAF scores than HC/nc. The percentage of tobacco users, the amount of units per week, and length of use were not different between groups. In terms of cannabis use, no significant differences were observed between groups for age of first use or units per week, while a significantly longer length of use was observed in SCZ/c with respect to HC/c (U = 1.00; *p* < 0.05).

### 3.2. FTIR Spectrometry Principal Component Analyses

**Figure 1:**
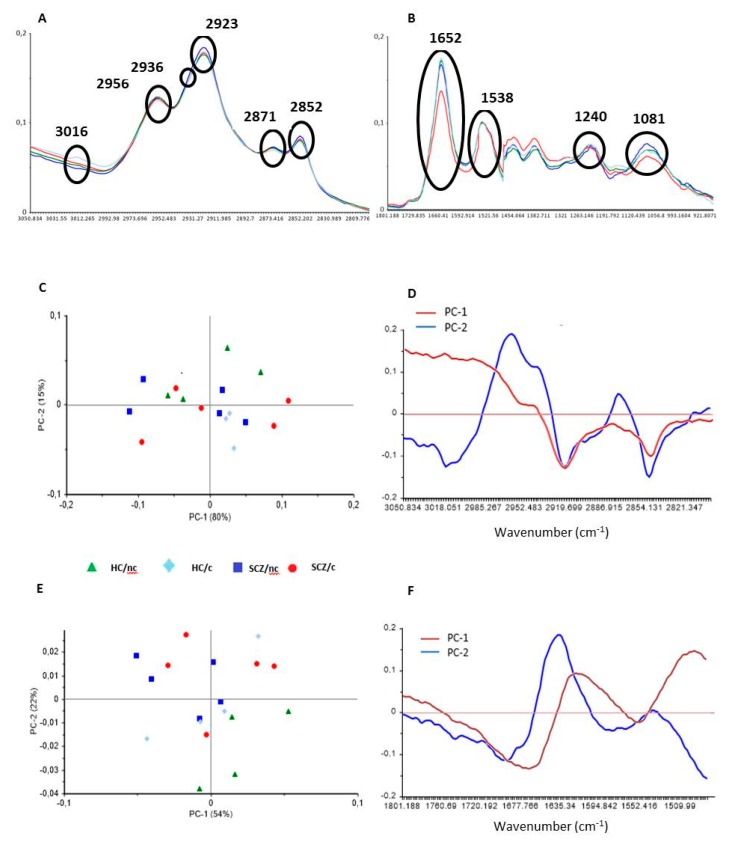
Mean FTIR of dried olfactory neuroepithelium (ON) cells from healthy controls non-cannabis users (HC/nc, green); healthy controls cannabis users (HC/c, light blue); schizophrenic patients non-cannabis users (SCZ/nc, dark blue) and schizophrenic patients cannabis users (SCZ/c, red). The main peaks are assigned for (**A**) the lipid region (3050–2800 cm^−1^), and (**B**) the protein fingerprint region (1800–900 cm^−1^). Contribution of the first two components in the principal component analysis (PCA) for lipids (3050–2800 cm^−1^) (**C**,**D**), and for proteins (1800–1480 cm^−1^) (**E**,**F**). In C, and E, symbols represent the mean values for each subject.

To study the biochemical changes underlying SCZ and how cannabis use could differentially modulate these alterations we averaged SR-FTIR spectra from each group for the lipid (Figure 1A) and protein (Figure 1B) fingerprint regions. Samples from 5 subjects in each group were prepared for measurements, although cells from one subject in the HC/c group did not survive. From each subject, we selected 20 to 32 cells, and for each cell we took two independent measures: one in the cytoplasm and another in the nucleus. The final sample size was as follows: HC/nc (*n* = 5 subjects × 25 cells = 246 measurements); HC/c (*n* = 4 subjects × 32 cells = 256 measurements); SCZ/nc (*n* = 5 subjects × 20 cells = 198 measurements); SCZ/c (*n* = 5 subjects × 27 cells = 268).

The PCA for the lipid region (3050 cm^−1^ to 2800 cm^−1^) is shown in Figure 1C. The first two components of each region of the spectra accounted for 80% (PC1) and 15% (PC2) of the total variability. For PC1 and PC2, a maximum was observed at ~2956 cm^−1^ corresponding to the asymmetric stretching vibration of CH_3_ (*ν*_asym_ CH_3_), and a minimum at ~2920 cm^−1^, indicating differences in the CH_2_ asymmetric stretching vibrations (*ν*_asym_ CH_2_). For PC2 one peak with maxima at ~2870 cm^−1^ was observed, and for both PC1 and PC2 a minimum peak at ~2844 cm^−1^ was detected, pointing to CH_3_ and CH_2_ symmetric stretching vibrations, respectively (Figure 1D). The PCA for the Amide I and II protein regions including the ester bound (1800 cm^−1^ to 1480 cm^−1^) is shown in Figure 1E. The first two components of each region of the spectra accounted for 54% (PC1) and 22% (PC2) of the total variability, respectively. The PC1 of protein bands around 1655 (Amide I) and 1545 cm^−1^ (Amide II) areas showed a minimum at ~1655 cm^−1^ (assigned to α helix secondary conformation of proteins), and a maximum at ~1620 cm^−1^ (mostly from cross-β-sheet structures). For PC2, a maximum at ~1635 cm^−1^ (β structure) and minimum at around ~1690 cm^−1^ (antiparallel β structure) were observed (Figure 1F). These data reveal differences in protein secondary structure between the different groups.

### 3.3. FTIR Spectrometry Analysis in the Lipid Region

**Figure 2:**
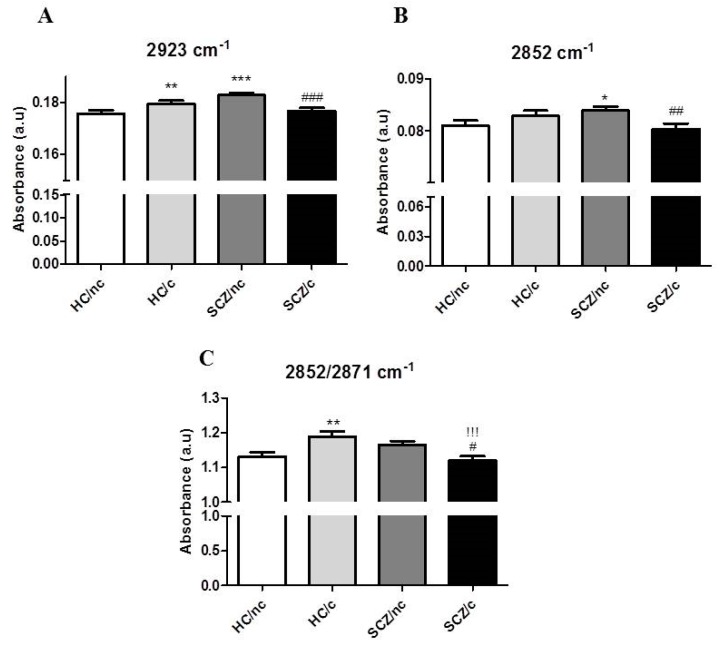
Major peak amplitudes related to the CH2 (**A**) 2923 cm-1, (**B**) 2852 cm-1 and (**C**) the ratio between 2852/2871 cm-1 in ON cells from healthy controls non-cannabis users (HC/nc); healthy controls cannabis users (HC/c); schizophrenic patients non-cannabis users (SCZ/nc) and schizophrenic patients cannabis users (SCZ/c). The data are expressed as means ± S.E.M. *p < 0.05; **p < 0.01; ***p < 0.001 vs. HC/nc. ^!^*p* < 0.05; ^!!^*p* < 0.01; ^!!!^*p* < 0.001 HC/c vs. SCZ/nc. ^#^*p* < 0.05; ^##^*p* < 0.01; ^###^*p* < 0.001 SCZ/nc vs. SCZ/c.

In the lipid region of the spectra, we analysed the amplitude of the most pronounced peaks at (2923 and 2852 cm^−1^), as well as, the ratio of symmetric CH_2_/CH_3_ vibrations (2952/2871 cm^−1^) after baseline correction and vector normalization (Figure 2). The peak at 2923 cm^−1^ is mainly related to long phospholipidic chains [31]. This band is also influenced by CH groups, which result from oxidation of CH_2_ groups to hydroxymethine groups, pointing to a possible oxidation effect [32]. Statistical analysis revealed that both HC/c (*p* < 0.01) and SCZ/nc (*p* < 0.001) showed significantly higher amplitudes than HC/nc. In addition, SCZ/c showed a significantly lower amplitude than SCZ/nc (*p* < 0.001), and no difference in amplitude compared to HC/nc (one-way ANOVA F(3,922) = 7.347; *p* < 0.001) (Figure 2A). The second most prominent contribution was at 2852 cm^−1^ (*ν*sym CH_2_), corresponding to the symmetric stretching vibration of CH_2_, arguing that CH_2_ group may show a disruption in some groups. The 2852 cm^−1^ peak has been shown to be a reliable indicator of the lipidic character of a cellular compartment since CH_2_ absorption increases with the presence of internal lipid membranes, such as those of the Golgi apparatus and the endoplasmic reticulum, and could also explain the lipid metabolic status of a cell [33,34]. A significant increase in the amplitude of this peak was found in SCZ/nc with respect to HC/nc (*p* < 0.05), while SCZ/c exhibited a significantly lower amplitude than SCZ/nc (*p* < 0.01) (one-way ANOVA F(3,922) = 2.971; *p* < 0.05), (Figure 2B). An increased ratio 2852/2871 cm^−1^ has been associated to a higher acyl chain unsaturation level, which is known to happen with lipid peroxidation [30]. A significant increase was observed in HC/c with respect to HC/nc, while a significant decrease in this ratio was found in SCZ/c with respect to SCZ/nc (*p* < 0.5) and to HC/c (*p* < 0.001) (one-way ANOVA F(3,922) = 5.157; *p* < 0.01) (Figure 2C).

**Figure 3:**
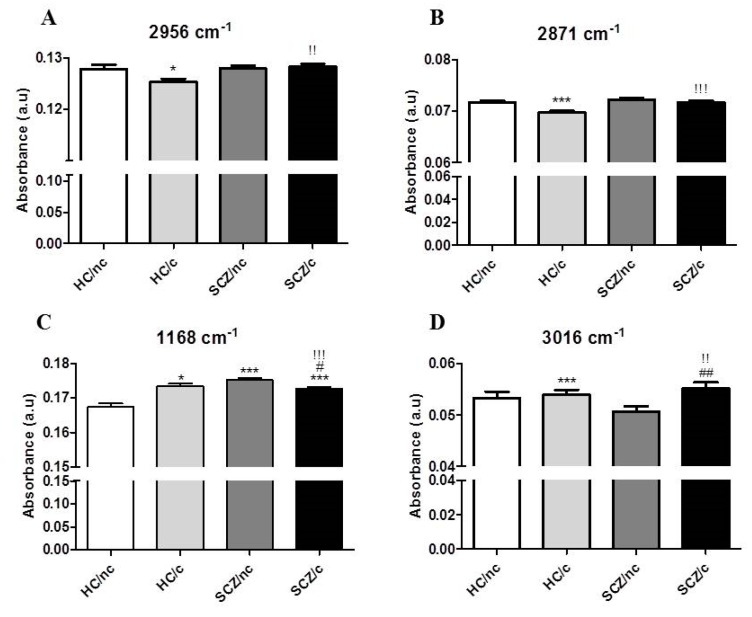
Major peak amplitudes related to the CH3 (**A**) 2956 cm^−1^, (**B**) 2871 cm^−1^, (**C**) the cholesteryl esters 1168 cm^−1^ and (**D**) the aromatic cys-alkelene (HC=CH) group 3016 cm^−1^ in ON cells from healthy controls non-cannabis users (HC/nc); healthy controls cannabis users (HC/c); schizophrenic patients non-cannabis users (SCZ/nc) and schizophrenic patients cannabis users (SCZ/c). The data are expressed as means ± S.E.M. * *p* < 0.05; ** *p* < 0.01; *** *p* < 0.001 vs. HC/nc. ^!^ *p* < 0.05; ^!!^ *p* < 0.01; ^!!!^ *p* < 0.001 HC/c vs. SCZ/nc. ^#^ *p* < 0.05; ^##^ *p* < 0.01; ^###^ *p* < 0.001 SCZ/nc vs. SCZ/c.

The peak at 2956 cm^−1^ band (Figure 3A) is assumed to show the asymmetric stretching vibration of CH_3_ groups in fatty acids, phospholipids and cholesterol esters, describing the state of the plasma membrane composition [35,36]. Statistical analysis reported a decrease in the amplitude of this peak in HC/c with respect to HC/nc (*p* < 0.05), and a significant increase in SCZ/c as compared to HC/nc (*p* < 0.01) (one-way ANOVA F(3,922) = 2.927; *p* < 0.05). The 2871 cm^−1^ band is also related to phospholipids (Figure 3B), but it is influenced by protein CH_3_ groups [33]. Post-hoc comparisons revealed a significant decrease in amplitude in HC/c compared to HC/nc (*p* < 0.001), and in SCZ/c with respect to HC/c (*p* < 0.001) (one-way ANOVA F(3,922) = 7.347; *p* < 0.001). In addition, we also analysed the fingerprint for the lipid region in order to check the asymmetric stretching vibrations of the cholesteryl esters and triglycerides (CO-O-C), which is associated to the 1170 cm^−1^ peak [37]. Our sample showed a slight shift in this peak, moving to 1168 cm^−1^ (Figure 3C). Here, the LSD post-hoc test showed significant increased amplitudes with respect to HC/nc in HC/c (*p* < 0.05), SCZ/nc (*p* < 0.001), and SCZ/c (*p* < 0.001). However, SCZ/c showed a significantly lower amplitude than HC/c (*p* < 0.001), and SCZ/nc (*p* < 0.05) (one-way ANOVA F(3,922) = 24.724; *p* < 0.001). Together, these changes point to a differential effect of cannabis in SCZ patients and in healthy controls.

Other narrow peaks that did not account for the saturated lipids spectra were found in the second component, such as the 3016 cm^−1^ peak (Figure 3D), which corresponds to the stretching vibration of the aromatic (cys-alkene (HC=CH)) group. This peak predominantly belongs to polyunsaturated acids, and its presence indicates an increase in the unsaturated and polyunsaturated lipids of the cell, which are related to many bioactive and lipid mediators [38]. Statistical analysis of the absorbance of this band revealed that between healthy non-cannabis users and healthy cannabis users (*p* < 0.001); between healthy cannabis users and SCZ cannabis users (*p* < 0.01); and between SCZ non-cannabis users and SCZ cannabis users (*p* < 0.01) (one-way ANOVA (F(3,922) = 11.212; *p* < 0.001).

### 3.4. FTIR Spectrometry Analyses of DNA Methylation

**Figure 4:**
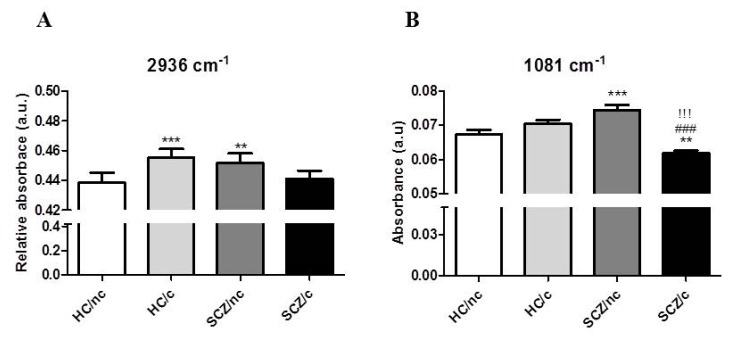
Peak amplitudes for the methylation related peak 2936 cm-1 (**A**) and the symmetric stretching vibrations of the phosphate group (1081 cm-1) (**B**) in ON cells from healthy controls non-cannabis users (HC/nc); healthy controls cannabis users (HC/c); schizophrenic patients non-cannabis users (SCZ/nc) and schizophrenic patients cannabis users (SCZ/c). The data are expressed as means ± S.E.M. * *p* < 0.05; ** *p* < 0.01; *** *p* < 0.001 vs. HC/nc. ^!^ *p* < 0.05; ^!!^ *p* < 0.01; ^!!!^ *p* < 0.001 HC/c vs. SCZ/nc. ^#^ *p* < 0.05; ^##^ *p* < 0.01; ^###^ *p* < 0.001 SCZ/nc vs. SCZ/c.

Moreover, another narrow peak was identified thanks to the PCA around the 2936 cm^−1^ (Figure 4A). This peak was first found to be a shoulder peak from the 2923 cm^−1^ band, which could reflect variations in the methylation levels of DNA and histones [31,35]. After applying the Norris GAP derivative approach, we found a significant increase in peak amplitude in HC/c (*p* < 0.001), and in SCZ/nc (*p* < 0.01) as compared to HC/nc, while no significant differences were found in SCZ/c with respect to HC/nc (one-way ANOVA F(3,922) = 7.199; *p* < 0.001). Finally, the intensity of the phosphate bands was analysed further after correcting and unit vector normalizing spectra of the nucleic acid region (1480 cm^−1^ to 960 cm^−1^). Significant differences were observed for both the 1240 cm^−1^ and the 1081 cm^−1^ peak (Figure 4B), which correspond to the asymmetric and the symmetric stretching vibrations of the phosphate group (PO_2_^−^) respectively. Differences between groups were only found in the phosphate symmetric stretching vibration intensity band (around 1081 cm^−1^) (one-way ANOVA F(3,922) = 17,503; *p* < 0.001), and the post-hoc test reported differences between healthy non-cannabis users and SCZ non-cannabis users (*p* < 0.001) and SCZ cannabis users (*p* < 0.01); between healthy cannabis users and SCZ cannabis users (*p* < 0.001); and between SCZ non-cannabis users and SCZ cannabis users (*p* < 0.001).

### 3.5. FTIR Spectrometry Analysis in the Protein Region

**Figure 5:**
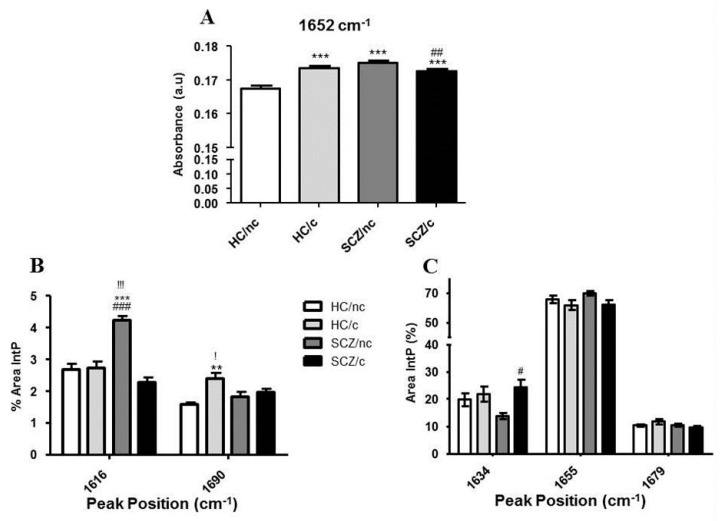
Major peak amplitudes related to the protein signature in ON cells from healthy controls non-cannabis users (HC/nc); healthy controls cannabis users (HC/c); schizophrenic patients non-cannabis users (SCZ/nc) and schizophrenic patients cannabis users (SCZ/c). (**A**) Peak amplitudes for the Amide I peak (1652 cm^−1^). (**B**,**C**) Amide I area FTIR spectra deconvolution expressed as the percentage of the integrated area (%) of the protein secondary structures related to their centred peak positions. (**B**) Side chains/cross β-sheet (1616 cm^−1^), and antiparallel β-sheet (1690 cm^−1^). (**C**) β-sheet/unordered (1634 cm^−1^), α-helix (1655 cm^−1^), and turns & loops (1679 cm^−1^). The data are expressed as means ± S.E.M. * *p* < 0.05; ** *p* < 0.01; *** *p* < 0.001 vs. HC/nc. ^!^ *p* < 0.05; ^!!^ *p* < 0.01; ^!!!^ *p* < 0.001 HC/c vs. SCZ/nc.

The amplitude of the amide I peak (1652 cm^−1^) (Figure 5A) was found to be significantly higher in HC/c (*p* < 0.001), SCZ/nc (*p* < 0.01), and SCZ/c (*p* < 0.001) as compared to HC/nc. However, the amplitude of this peak was decreased in SCZ/c with respect to SCZ/nc (*p* < 0.01) (one-way ANOVA F(3,950) = 17.477; *p* < 0.001). Following deconvolution of bands 1700 cm^−1^ to 1600 cm^−1^, several different hidden peaks in the amide I region were found, corresponding to different secondary protein conformations (Figure 5B,C). Differences of the major secondary protein structures were analysed by comparing the percentage of integrated area (%) of each peak. The secondary major structures found in the deconvoluted area were: (i) side chains and cross β-sheet with a centre peak at 1616 cm^−1^; (ii) β-sheet with a centre peak at 1634 cm^−1^; (iii) α-helix chains with a centre peak at 1655 cm^−1^; (iv) different turns and loops secondary structures with a centre peak at 1679 cm^−1^; and (v) antiparallel β-sheet with a centre peak at 1690 cm^−1^. For the integrated area of the side chains/ cross β-sheet structures (1616 cm^−1^), statistical analysis showed a significant increase in SCZ/nc with respect to HC/nc (*p* < 0.01), to HC/c (*p* < 0.01), and to SCZ/c (*p* < 0.001) (one-way ANOVA F(3,17) = 22.95; *p* < 0.001) (Figure 5B). Furthermore, we found differences in the antiparallel β-sheet integrated area (1690 cm^−1^), which is a characteristic of protein oligomer aggregates. The results revealed a significant increase in HC/c with respect to HC/nc (*p* < 0.01), and to with respect to SCZ/c (*p* < 0.05) (one-way ANOVA F(3,17) = 6.648; *p* < 0.01) (Figure 5B). Statistical analysis of the intramolecular β- sheet peak (1634 cm^−1^) revealed that SCZ/c showed a larger area than SCZ/nc (*p* < 0.05) (one-way ANOVA F(3,17) = 3.86; *p* < 0.05) (Figure 5C). No significant differences between groups were found for the areas of peaks 1655 cm^−1^ and 1679 cm^−1^.

## 4. Discussion

Several studies have investigated the lipidomic and proteomic profiles in blood serum, peripheral tissue, and post-mortem brains of SCZ patients [7,8,10], as well as in healthy cannabis users [39,40]. However, up to now, there is no information as to the modulation of these parameters by chronic cannabis use in patients with SCZ. Here, we applied SR-FTIR spectroscopy in ON cells to study alterations in lipid, fingerprint area, and protein composition related to SCZ and their modulation by cannabis use. In this study, we report similar alterations in ON cells of SCZ/nc and in HC/c, including disruption in lipid composition, increased lipid peroxidation, changes in protein secondary structure, and an increase in DNA and histone methylation. Importantly, our results suggest that cannabis use may prevent these alterations in SCZ patients.

Thanks to their low invasiveness and high scalability, ON cell models have been put forward as promising surrogates to explore neuronal biomarkers in SCZ, providing a unique opportunity to unveil novel potential molecular targets for diagnosis and treatment [19]. To our knowledge, this is the first study applying SR-FTIR spectroscopy in ON cells to study the macromolecular alterations in SCZ. The use of SR-FTIR spectroscopy to investigate lipid and protein profiles in ON cells represents an advantage over other proteomic or lipidomic methods, since it provides the ability to observe the interior of cells directly, without labelling, at micrometres resolution scale. Importantly, it also allows determination of the intracellular distribution of different elements in their native form, as opposed to purification-based methods, which cause significant alterations of labile structures.

In this study, SR-FTIR spectroscopy showed an increase in the amplitudes of the stretching vibration bands of CH_2_ (2923 cm^−1^ and 2852 cm^−1^) in ON cells of SCZ/nc and HC/c with respect to HC/nc, implying that the lipid chains in the cellular membranes are more disordered [41] in these two groups. Both the 2852 cm^−1^ peak and the ratio between the values of 2852 cm^−1^/2871 cm^−1^ peaks, are solid indicators of the internal membrane compartment formation/recycling status [32,34]. Indeed, when their values show a broadening toward higher amplitudes, the formation and presence of internal lipid membranes also show an increase, pointing to an oxidative state. These alterations reveal an increase in internal membrane compartments, which leads to an increased lipid metabolism and peroxidation and toxicity [32,42]. This is observed thanks to the CH_3_:CH_2_ ratio. While CH_3_ groups are distributed in the inner and hydrophobic region of lipid bilayer membranes, such as plasma membrane, endoplasmic reticulum or Golgi apparatus, CH_2_ groups are distributed along the hydrophilic region of these membranes. Since the plasma membrane is thicker than all the rest of internal membranes, it usually shows a higher CH_3_ group contribution. Thus, the CH_3_ relative contribution in the internal membranes decreases, while the CH_2_ relative contribution increases [32]. This allowed us to check the membrane status of ON cells, indicating an increased renewal membrane rate. In accordance, the observed increases in 2923 cm^−1^ peak amplitude in HC/c and SCZ/nc could also be explained by the oxidation of CH_2_ to hydroxymethyl groups [43], supporting the presence of oxidative stress in ON cells of these subjects. Given that oxidative stress is primarily caused by lipid peroxidation, we assume that lipid metabolism is also increased in ON cells from both HC/c and SCZ/nc. Additionally, HC/c show specific disturbances in the amplitudes of the stretching vibration of CH_3,_ 2956 cm^−1^ and 2871 cm^−1^, with respect to HC/nc. In similar terms, both SCZ/nc and HC/c showed an increase in the amplitudes related to the stretching vibrations of the cholesterol esters (1170 cm^−1^) with respect to controls. Thus, these results suggest an impoverishment in membrane lipid composition and diversity, as well as, an imbalance in the normal ratio between fatty acids:phospholipids:cholesterol esters [32]. Hence, there is an increase of cholesterol esters and triglycerides in detriment of a decrease in the phospholipid normal balance of the plasma membrane. These data show major alterations in lipid metabolism in HC/c, which are consistent with previous findings showing that cannabis use leads to a prominent increase in lipid peroxidation in isolated mitochondria extracted from rat brain [40]. Also, the disturbances found in SCZ/nc agree with data reporting increased pro-oxidative markers in blood plasma of SCZ patients [9]. Importantly, this increased oxidative state was not observed in ON cells from SCZ/c. In contrast, an increase in the 3016 cm^−1^peak was observed in all subjects that smoke cannabis (HC/c and in SCZ/c), indicating an increase in long-chain polyunsaturated lipid mediators, such as docosahexaenoic acid (DHA), arachidonic acid (ARA), or endocannabinoids (AEA and 2-AG) [38]. Endocannabinoids have complex actions depending on which receptors or brain structure, they target, including modulation of mood and memory processes, as well as neuroinflammation and energy metabolism homeostasis [44]. In frequent cannabis users, lower levels of AEA in cerebrospinal fluid, and higher levels of 2-AG in serum were observed as compared to infrequent cannabis users, and higher levels of AEA were associated with a lower risk of psychotic symptoms following cannabis use [45]. Accordingly, AEA levels in cerebrospinal fluid of acute SCZ patients are increased, and correlate negatively with psychotic symptoms [46], pointing to a protective role of AEA in cannabis users and SCZ patients

Additionally, our results also support the existence of an increase in DNA and histone methylation in ON cells of SCZ/nc and HC/c. The peak situated in the 2936 cm^−1^ band corresponds to CH_3_ groups from DNA and histone methylation [31], which is increased in some brain areas that are highly affected by SCZ neuropathology. This increase has been mostly reported as an increase of DNA methyltransferases in GABAergic neurons of SCZ patients [47]. In contrast, SCZ/c show similar levels of DNA and histone methylation than HC/nc, suggesting that cannabis use in SCZ patients could lead to a switch towards stable levels of DNA and histone methylation. Further studies using Raman scattering of our spectra may show more explicit changes in methylation.

Similarly, the protein region spectra analyses revealed major alterations in protein secondary structures in SCZ/nc and HC/c. First, we found a two-fold increase in the integrated area of the cross-β-sheet structures (centred at 1616 cm^−1^) specifically in SCZ/nc, which may be associated with protein fibril aggregates in the cells. In this sense, there is evidence showing that SCZ patients exhibit an accumulation of *Disc1* (Disrupted-in-schizophrenia 1) aggregates [6]. Interestingly, cannabis use prevents this effect in SCZ/c. In fact, others have reported how some protein aggregates rich in cross β-sheet structures are generated by an excess of oxidation, such as the β-amyloid aggregates found in post-mortem brains of Alzheimer patients [48,49]. Thus, our data showing an increase in oxidative stress in ON cells from SCZ patients could be connected with protein misfolding. On the other hand, we found an increase in antiparallel β-sheet integrated area (centred at 1690 cm^−1^) in HC/c, indicating the presence of aggregates or oligomers in these cells [50,51].

Together, our data suggest that cannabis use may induce differential effects in the macromolecular profile of SCZ patients and HC subjects. These opposite effects may be due to a differential basal state of the endocannabinoid system in both groups. Indeed, dysregulations in endocannabinoids and cannabinoid receptors have been reported in SCZ patients [52,53,54]. Also, differential effects of gene x environment interactions within the endocannabinoid system [55], or epigenetic changes in SCZ [56,57,58] versus HC subjects could be contributing to the differences observed. Finally, we cannot rule out that an interaction between cannabis use and antipsychotic treatment in SCZ patients may have participated in the observed effects. Thus, further studies are needed to understand the biological processes underlying the specific effects of cannabis in SCZ, and to assess which component of cannabis mediates these effects.

Our study has advantages and limitations. One advantage is the use of ON cell models that represent a highly novel substrate closely related to the central nervous system to explore biomarkers of neuropsychiatric disorders such as SCZ. Second, the evaluation of cannabis effects in separate cohorts of healthy controls and SCZ patients is highly relevant considering the lack of studies comparing these populations. Finally, the use of synchrotron-based FTIR spectroscopy to investigate lipid and protein profiles in human cells provides maximum resolution measurements. The limitation of our study was the small number of subjects per group. However, we maximised the number of measurements taken in each sample in order to obtain less variability in each group, and more statistical power. In agreement, the results obtained showed very tight standard errors, and revealed significant differences between groups. Nevertheless, additional studies with more subjects will be required to further corroborate these findings.

In conclusion, we report that ON cells from SCZ patients show prominent alterations in their lipid profile, secondary protein structures, and DNA and histone methylation as compared to control subjects. Importantly, our data revealed that cannabis use increases lipid peroxidation and lipid membrane disruption in HC/c, but reduces these detrimental parameters in SCZ/c. In addition, we found that SCZ/nc show a specific significant increase of cross β-sheet structures with respect to HC/nc, indicating a major change in protein organization and oxidation status, but this effect was not observed in SCZ/c. On the other hand, HC/c show a specific increase in antiparallel β-sheet structures with respect to HC/nc, pointing to an increase in aberrant protein aggregation or oligomers in these subjects. Moreover, we report and increase in DNA and histone methylation in SCZ/nc and in HC/c as compared to HC/nc, but not in SCZ/c. These findings indicate that cannabis may induce opposite effects in lipid, protein, and DNA methylation in HC, and in SCZ patients.

## Figures and Tables

**Table 1:** Demographic, socioeconomic, and functional characteristics of the sample.

	Healthy Controls (Non-Cannabis Users)	Healthy Controls (Cannabis Users)	Schizophrenia Patients (Non-Cannabis Users)	Schizophrenia Patients (Cannabis Users)
Age (years) ^†^	30 (27.5–36.5)	29 (22.5–41-5)	39 (26.5–46-5)	41 (36–47)
Gender; male (%)	2 (40.0)	4 (100.0)	2 (40.0)	5 (100.0)
BMI (kg/m^2^) ^†^	21.8 (19.9–25.37)	20.2 (12–25.35)	30.1 (23.54–41.45)	25.35 (24.1–29.23)
History of nasal trauma/surgery; n (%)	0 (0)	1 (25)	1 (20)	1 (20)
History of rhinitis; n (%)	1 (20)	0 (0)	0 (0)	0 (0)
Tobacco Use				
Users–n, (%)	5 (100)	2 (50)	3 (60)	5 (100)
Units per week ^†^	49 (22.5–122-5)	21 (14–28)	140 (105–210)	140 (87.5–210)
Length of use (years) ^†^	12 (8–16.5)	17	9 (4–16)	23 (15.5–33.5)
Cannabis Use				
Age first use (years) ^‡^	n/a	17 (2.16)	n/a	17.2 (3.00)
Units per week ^†^	n/a	5.5 (3.25–17.5)	n/a	21 (6.5–31.5)
Length of use (years) ^†^	n/a	6 (1–13)	n/a	22 (16–31.5) *
Hollingshead-Redlich Score ^†^	45.5 (38.75–47)	57 (49–62.5)	52 (16.5–60,5)	52 (41–56)
Global Assessment of Functioning (GAF) ^†^	100 (97.5–100)	100 (92.5–100)	65 (60–75) *	65 (60–65) *

For continuous, non-normally distributed variables (†) results are reported in median (first quartile–third quartile) and provided statistics refer to the Kruskal–Wallis or Mann–Whitney tests followed by pairwise post-hoc test when appropriate. For continuous, normally distributed variables (‡) results are reported in mean (standard deviation). BMI = Body Mass Index; n/a = not applicable; * *p* < 0.05 vs. healthy controls.
